# Developing good practice indicators to assist mental health practitioners to converse with young people about their online activities and impact on mental health: a two-panel mixed-methods Delphi study

**DOI:** 10.1186/s12888-022-04093-w

**Published:** 2022-07-19

**Authors:** Lucy Biddle, Raphael Rifkin-Zybutz, Jane Derges, Nicholas Turner, Helen Bould, Felicity Sedgewick, Rachael Gooberman-Hill, Paul Moran, Myles-Jay Linton

**Affiliations:** 1 Population Health Sciences, Bristol University Medical School, Canynge Hall, 39 Whatley Road, Bristol, UK; 2 The National Institute for Health Research Applied Research Collaboration West (NIHR ARC West) at University Hospitals Bristol and Weston NHS Foundation Trust, Bristol, UK; 3 Centre for Academic Mental Health, Bristol University Medical School, Oakfield House, Oakfield Road, Bristol, UK; 4 South London and the Maudsley NHS Foundation Trust, Bethlem Royal Hospital, Beckenham, UK; 5 Medical Research Council Integrative Epidemiology Unit, University of Bristol, Bristol, UK; 6 Gloucestershire Health and Care NHS Foundation Trust, Gloucester, UK; 7 School of Education, University of Bristol, Bristol, UK; 8 Bristol University Medical School, Learning and Research Building, University of Bristol, Southmead Hospital, Level 1, Bristol, UK; 9 Biomedical Research Centre at University Hospitals Bristol and Weston NHS Foundation Trust, Bristol, UK

**Keywords:** Children, Adolescent, Young people, Mental disorder, Internet, Social media

## Abstract

**Background:**

Online activity has been linked to poor mental health in children and young people, particularly those with existing vulnerability who may inadvertently or otherwise access harmful content. It is suggested health and social care practitioners should address online activity during mental health consultations, but guidance about acceptable or effective ways to do this is lacking. This study sought to derive good practice guidance to support mental health practitioners to engage young people in conversations about their online activities and impact on mental health.

**Methods:**

A mixed-methods Delphi (consensus) study was conducted with a panel of mental health practitioners (*n* = 21) and a panel of young people (*n* = 22). Practitioners worked with children or young adults in the UK, mostly in statutory services (80.9%), in varied clinical roles, with 2 – 30 years of experience and most were female (87.5%). Young people were mostly female (77.3%), 13—22 years old, reported varied mental health diagnoses and had sought help from services. Across 3 rounds, panellists completed questionnaires which involved rating agreement with statements and answering open-ended questions. Iterative analysis informed subsequent questionnaire content. The percentage of participants rating their level of agreement with each statement was calculated. The threshold for inclusion as a good practice indicator (GPI) was 75% across both panels. Thematic analysis was used for free-text data.

**Results:**

Twenty-seven GPIs emerged covering ‘who’ (which young people) should be asked about online activities, ‘when’, ‘what’ should be discussed, and with what ‘outcome’. Panels agreed conversations should be initiated with all young people from first meeting and regularly thereafter, with ‘red flags’ indicating a conversation may be pertinent. Core topics were identified with additional areas for patients presenting with disordered eating or self-harm. Panels emphasised conversations should be fluid, normalised, and encourage reflection and self-awareness.

**Conclusions:**

Mental health practitioners could empower young people to exercise agency in relation to online safety and capitalise on positive features. Findings also identify training needs for practitioners. Further research should explore real-world application of the GPIs and transferability to underrepresented groups within our panels, such as males and younger children. Ethnicity and deprivation were not recorded.

**Supplementary Information:**

The online version contains supplementary material available at 10.1186/s12888-022-04093-w.

## Background

Social media and internet use have been linked to poor mental health in children and young people by a significant body of empirical literature of varying study designs [1]. Areas of concern include sense of self, body image and disordered eating [2], socioemotional functioning [3], sleep disturbance [4], and self-harm and suicidality [5, 6]. Yet, positive impacts are also described, such as improved social connectedness and support, and there is recognition that effects may vary according to the characteristics and circumstances of the young person, including their existing vulnerability [7]. This is of import given the ubiquity of device ownership amongst children, adolescents and young adults.

Actions to tackle online harm focus on legislation to increase the responsibilities of platforms and providers and school-based online safety education [8]. A role for health and social care practitioners in addressing online activity during consultations has also been intimated [1, 9, 10], with suggestion this could involve harm reduction by recommending reduced usage, encouraging parental involvement, discussing risks and sleep hygiene, use of motivational interviewing to reduce excessive usage, promotion of digital citizenship and incorporation of online safety into crisis planning. In 2019, the UK Royal College of Psychiatrists [11] recommended psychiatrists should routinely ask about technology use when undertaking mental health assessments with children and young people.

Preliminary evidence indicates asking patients about their online activity may be clinically beneficial. For instance, in a qualitative study, psychiatric liaison clinicians reported that exploring online behavior with patients presenting following self-harm could contribute to assessment of risk by deepening understanding of the individual, and identifying motivations behind suicidal behavior, active suicidal intent and disguised requests for help [12]. However, some clinicians were reticent to ask in case this prompted patients to access ‘bad’ content and some sought guidance about how to respond to disclosures of harmful use. While it is likely that such conversations increasingly take place within consultations, guidance about how to conduct these is lacking. In our scoping research (unpublished observations: Rifkin-Zybutz, Derges, Biddle et al.), 93% of 86 child and adolescent mental health practitioners surveyed reported having no access to a protocol to guide discussion around online activities with young patients, 76% had received no training or guidance, only 54% covered this topic routinely and 71% expressed a wish for training. Alongside these data, many adolescents reported a lack of satisfaction where such conversations had taken place as part of their care due largely to a sense of feeling judged. Despite this, 92% of practitioners and 68% of young people surveyed agreed health and social care practitioners should contribute to ensuring the safety of young people online and 73% of practitioners believed exploring online use should form an essential part of risk assessment.

This study aimed to identify good practice indicators to inform development of guidance to assist mental health practitioners to engage young people in acceptable, safe and useful conversations about their online activities and how these impact on their mental health. Consensus was achieved by using a two-panel, three-stage Delphi method, drawing upon the expertise of child and adolescent mental health practitioners and young people with lived-experience of mental health problems and service use.

## Methods

This study was part of a wider programme of research investigating young people’s digital technology in the context of mental health consultations and management of risk. The Delphi method is used to systematically determine expert consensus. It is of proven utility in mental health research [13] and has been used to explore areas similar to that considered here [14]. We used a mixed-methods design. This involved: 1) using findings from prior engagement research to develop an initial Delphi questionnaire; 2) three iterative rounds of quantitative and qualitative data collection across two panels (mental health practitioners and young people); 3) development of good practice indicators.

### Questionnaire development

The Delphi questionnaire was informed by research conducted in the UK with young people aged 13–24 years and mental health practitioners who worked with children or young people. This comprised a series of online surveys, focus groups and semi-structured interviews to clarify the importance of the topic to both groups and to identify dimensions to investigate within the Delphi. The resultant questionnaire was shared with three practitioners and a young person’s research advisory group member, who suggested refinements to improve clarity and ensure the language used was accessible. This process is detailed in an additional file [Additional File 1].

### The Delphi process

A panel of practitioners (PP) and a panel of YP (YPP) were recruited by advertising on Twitter, via UK-based third sector organisations, within networks established during our engagement phase, and through snowball sampling. Potential participants completed a brief online screening questionnaire and, if eligible, were invited by email to take part. Eligible practitioners were health or social care professionals currently working in young people’s mental health services. Eligible YP were aged 12–24 years with experience of using mental health services. Recruitment was open for 6 weeks. Participants became panel members upon completing questionnaire 1.

All questionnaires were administered online and completed anonymously. Informed consent was provided at the start of questionnaire 1, including parental consent for under 16s. The Round 3 questionnaire was sent to all panel members irrespective of whether they completed Round 2. Questionnaires contained a mix of i) statements to be rated for agreement using a 5-point Likert scale (strongly disagree, disagree, neither agree nor disagree, agree, strongly agree) and ii) open-ended questions and free-text boxes for further comments. The questionnaires for Rounds 2 and 3 differed by panel according to the specific views previously obtained in each (below). The entire Delphi took place between December 2020 and April 2021. A shopping voucher was offered as a gratuity on completion of each questionnaire.

Analysis took place after each round to enable an iterative approach, the next round being used to clarify, develop and refine emerging perspectives. Findings for each panel were analysed separately (not pooled) to highlight areas of difference and similarity. Quantitative data were analysed in Stata v.16. The number and percentage of participants rating their level of agreement with each statement at each point on the scale was calculated to examine the level of similarity among participants (consensus). Guided by other studies [15], statements were considered to have reached consensus when 75% or more of the panel either agreed (rated statement as strongly agree or agree) or disagreed (rated statement as strongly disagree or disagree). Stability was considered for items repeated over two rounds and not reaching the consensus threshold. The percentage shift between rounds was calculated across the three main categories of response (strongly agree/ agree; unsure; disagree/ strongly disagree). Items with a shift of less than 10% across all categories were considered to have reached ‘stable non-consensus’. Qualitative free-text data were coded inductively using thematic analysis to identify themes. LB and JD coded independently then compared findings. Similar ideas were grouped together into conceptual categories.

In Round 2 and 3 questionnaires:

i) Statements that had reached consensus were not repeated. Statements without consensus were repeated with the panel’s previous round of results shown as aggregate percentages expressing current agreement, disagreement and uncertainty, and a summary of any relevant free-text data expressing arguments for and against the statement;
ii) Statements showing stable non-consensus across Rounds 1 and 2 were removed from Round 3 on the basis this indicated the panel could form no clear view;
iii) Open-ended questions were added to gain further detail around accepted statements, or nuance where consensus could not be reached within or across panels. Equivalent questions were asked of each panel;
iv) New statements were added following qualitative analysis of free-text and included for both panels to ensure they were rated by both groups.

### Identification of good practice indicators

Agreement rates for each statement were tabulated and compared across panels. Statements that reached consensus in both panels were progressed for possible inclusion as Good Practice Indicators (GPIs). Where statements were agreed early in the Delphi and then superseded by more precise follow-on statements, only the latter were retained. Lists of statements—such as ‘red flags’ indicating when practitioners should ask about online activities—were grouped and presented as a single overarching statement with accompanying sub-items. Other related statements were also merged. Statements about practitioner support needs were not regarded as GPIs and were noted separately.

Statements reaching consensus in one panel but not the other were then considered. These were identified as ‘near-misses’ where the second panel expressed a majority trend in the same direction as the panel with consensus, or as ‘conflicts’ where the majority trend was opposing. The strength of the near-miss or conflict was then ranked according to the majority percentage in the second panel as low (50–59%), medium (60–69%), or high (70–74%). Information from near misses and conflicts, and insights from free-text data, were used to inform a supporting commentary.

## Results

From a total pool of 44 practitioners and 40 young people, 41 and 38 respectively were eligible and invited to participate. We recruited 21 practitioner panel (PP) members and 22 young people panel (YPP) members (Table 1). All were based in England or Wales covering urban and rural settings. Practitioners were mostly females (87.5%) working in the statutory sector (80.9%) but represented a range of clinical roles and years of experience. The YPP were diverse in age, diagnoses and experience of service use. Three-quarters were female. Ethnicity and deprivation were not recorded. Retention rates were: 76% (PP) and 95% (YPP) in Round 2; 71% (PP) and 73% (YPP) in Round 3.

**Table 1:** Panel characteristics

Practitioner panel (PP) (*n* = 21)		Young People Panel (YPP) (*n* = 22)	
**Gender, n (%)**		**Gender, n (%)**	
Male	3 (14.3)	Male	4 (18.2)
Female	18 (85.7)	Female	17 (77.3)
**Years of relevant practice**		Non-binary	1 (4.5)
Range	2 – 30	**Age (years)**	
Median	8	Range	13 - 22
IQR	14	Median	17.5
**Clinical role, n (%)**		IQR	4
Psychiatrist	9 (42.9)	**Self-reported diagnosis**^**a**^**, n (%)**	
Paediatrician	1 (4.8)	Anxiety	12 (54.5)
Psychologist	4 (19.0)	Depression	10 (45.4)
Nurse	4 (19.0)	Eating disorder	7 (31.8)
Student/ school counsellor	2 (9.5)	Psychosis	1 (4.5)
Psychotherapist	1 (4.8)	Obsessive compulsive disorder	2 (9.1)
**Sector, n (%)**		ADHD	1 (4.5)
Statutory (NHS Child & Adolescent Mental Health Services)	17 (80.9)	Post-traumatic stress disorder	3 (13.6)
Statutory (Not specified)	1 (4.8)	Emotional dysregulation	1 (4.5)
Education (University/ school)	2 (9.5)	Self-harm	1 (4.5)
Private sector	1 (4.8)	Borderline Personality Disorder	1 (4.5)
		**Services ever used**^**a**^**, n (%)**	
		Statutory (NHS Child & Adolescent Mental Health Services)	15 (68.2)
		Statutory (GP only)	1 (4.5)
		Private therapist	6 (27.3)
		Charity-based counselling services	2 (9.1)
		School counsellor	2 (9.1)

^**a**^categories not mutually exclusive

Each panel rated 135 statements; 76 introduced in Round 1 and 59 added across Rounds 2 and 3. Statements are detailed with percentage agreements in an additional data file [Additional File 2]. Four core domains were explored:

- ‘who and when’ (28 statements) - which young people should be asked by practitioners about their online activities, how frequently, and in what circumstances?
- ‘how’ (30 statements) - most appropriate ways of starting and conducting conversations about online activities to maximise engagement and minimise blame or stigma;
- ‘what’ (61 statements) - what should be covered within conversations about online activity to enable practitioners to identify risk and support a young person’s mental health?;
- ‘outcomes’ (16 statements) - what should be done with the information from conversations, particularly where risky use is uncovered?.

The number of statements accepted, rejected, or without consensus by domain, round and panel are tabulated in an additional data file with commentary describing patterns across panels [Additional file 3]. There was strong overlap across panels, although the PP reached consensus on a greater number of items and were more likely to reject items than the YPP. The most contentious domain was ‘who and when’—the PP favouring a more inclusive approach, while members of the YPP expressed concerns about possible iatrogenic risks of discussing online content with some young people. Disagreements and uncertainties are detailed below.

### Good practice indicators

**Fig. 1:**
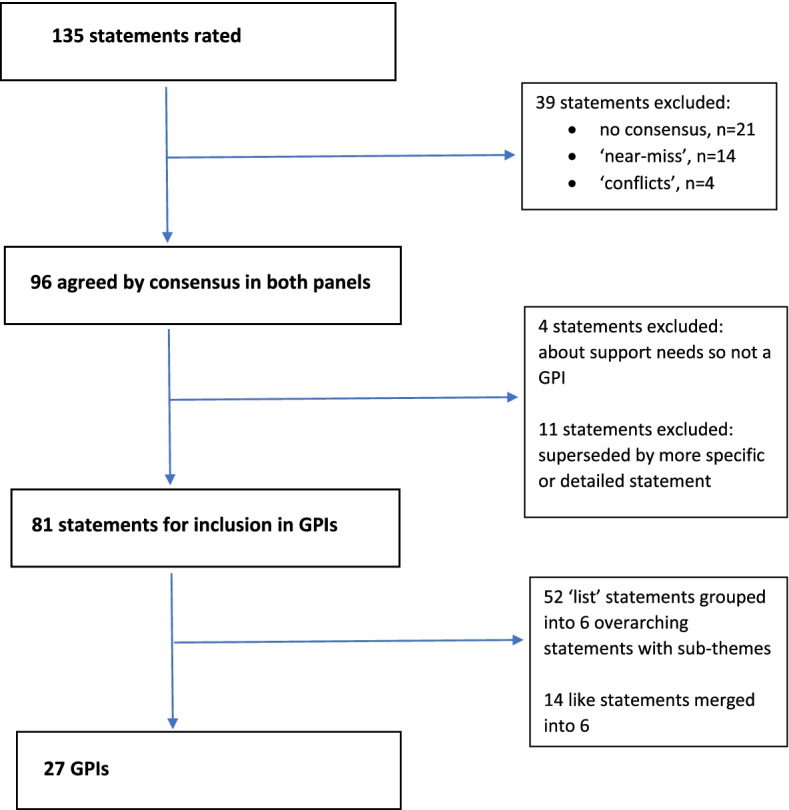
Flow chart showing creation of good practice indicators

Ninety-six statements were agreed by consensus in both panels and progressed for possible inclusion (Fig. 1).

These were organised into 27 Good Practice Indicators (GPIs) (Table 2).

**Table 2:** Good Practice Indicators for asking young people about online activities during mental health consultations

‘WHO’ to ask and ‘WHEN’ to ask	‘WHAT’ to ask – the content of conversations
1. All young people attending a mental health consultation should be asked about their online activities 2. Practitioners should ask young people about online activities at their first meeting and then at regular intervals 3. There are red flags that indicate circumstances (times/ young people) where it might be particularly necessary/ helpful to initiate a conversation (Table 3)	1. There is a set of key topics that it is important for clinicians to always ask about when exploring online activities (Table 4) 2. There is a set of key topics that clinicians should ask young people with disordered eating when exploring online activities (Table 4) 3. There is a set of key topics that clinicians should ask young people presenting with self-harm or suicidal thoughts when exploring online activities (Table 4) 4. Discussions about worrying online activity should usually include asking for the names of sites visited, descriptions of content created by the young person and details of participation in online groups 5. Asking about online activities should take the form of a deeper conversation in which clinicians encourage the young person to reflect on their behaviour patterns and the impacts of these (Table 5) 6. Adapted approaches may be necessary if asking younger age groups or young people with neurodevelopmental disorders
**‘HOW’ to encourage disclosure and ensure a non-judgemental approach**	**‘OUTCOMES’ – following up on conversations**
1. Discussion about online activities should be started spontaneously as part of the flow of conversation, with questions naturally embedded within broader topics rather than as a standalone item 2. Conversations should be supported by open-ended prompts 3. All young people should be offered an opportunity to discuss their online activities without their parent/ guardian being present 4. When discussing online activities, clinicians should be curious and ask questions 5. When discussing online activities, clinicians should be up-to-date in their knowledge of the online world and able to use up-to-date language	1. Online activity that causes concern should be flagged in notes so that it can be followed-up at other appointments 2. Clinicians should involve parents in conversations about a young person’s online activities if the young person is under 12-years-old 3. Clinicians should encourage the young person to be active in taking care of their own online safety 4. A clinician should not simply recommend stopping online activities but support the young person to engage with the online world in a more positive way 5. Aspects of online safety should be incorporated into treatment/ safety plans (Table 6) 6. When recommending apps, it is important for a clinician to offer several choices of app, always offer to demonstrate apps, and then follow-up on whether the recommendation was helpful or not
*Clinicians should:*	
6. Ask about positive aspects of online activities before addressing the negative 7. Always explain why they are asking the young person about the online activities 8. Always openly communicate understanding that online activities can be beneficial 9. Normalise online activities and acknowledge how commonplace online harm can be when discussing this with young people 10. Explicitly address fears of judgement or of ‘being in trouble’ when introducing questions about online activities 11. Explicitly discuss confidentiality and its limits when asking questions about online activity 12. Let young people know that they ask questions about online activity routinely during consultations	

### Included statements

It was agreed practitioners should ask all young people about their online activities (PP 95%, Round 1; YP 85%, Round 3); that this should happen at their first meeting (for instance, when taking a history or assessing risk) and should not be a ‘one-off’ conversation but take place regularly, as required. Agreed ‘red flags’ indicating a conversation may be particularly helpful to pursue focused on the presence of specific or changing patterns of device use (e.g. increase in notifications), presenting problems (e.g. bullying) or symptoms (e.g. sleep disturbance or suicidal thoughts) (Table 3).

**Table 3:** Red Flags indicating a conversation about online activities may be particularly helpful

Young person presents with:
• **Notable or changing patterns of device use**
Over-use, increase of notifications, device reliance, protectiveness of device, continually distracted by device (observed in session or concern reported by parent/carer)
**• Secrecy over device and online activities** (observed in session/ reported by parent/carer)
• **Negative self-image**
Concern about appearance and body image, unfavourable comparison with others
• **Signs of being isolated or withdrawn**
Withdrawing from friends/ family/ usual activities, spending more time alone
• **Self-harm behaviours or suicidal thoughts**
Especially changing methods of harm
• **Experience of bullying**
• **Sleep problems**
Changing pattern of sleep, excessive tiredness
• **Disordered eating**
• **Sudden change in presentation, disturbance of mood or behaviour**
Including drug and alcohol use
• **Signs of child sexual exploitation**
Including sexualised behaviours and past history of exploitation

Panels agreed such conversations should be: introduced spontaneously as part of the flow of conversation; open-ended and fluid rather than a tick-box assessment; contextualised with an explanation from the practitioner about why they are asking (for instance, by referencing possible risks); and that practitioners should approach conversations as a learner, being curious and prepared to ask questions to enhance their understanding of young people’s interactions with the online world. Further, to facilitate conversations, clinicians should have an up-to-date knowledge of the online world and be able to use up-to-date language. High levels of consensus (> 90%) were reached around practitioners explicitly addressing the young person’s potential fear of judgement and limits to confidentiality; providing opportunities to disclose without parents being present; normalising online activity and its discussion; and acknowledging that being online may also benefit mental health.

Topics that ‘should *always* be covered’ when discussing online activities were agreed, with tailored lists for patients presenting with disordered eating, self-harm or suicidal thoughts (Table 4). Where worrying online activity is uncovered, panels endorsed extending conversations to ask for the names of sites visited, descriptions of content created by the young person and details of participation in online groups. There was strong consensus (> 90%) that practitioners should also initiate reflective discussion, encouraging the young person to explore issues such as the thought processes and triggers underlying their online activities and the resulting impacts (Table 5). The statement that ‘practitioners should encourage young people to reflect on how they may cause harm to others online’ was introduced from YPP free-text and subsequently received 100% endorsement from this panel (PP, 79%), indicating this as a priority issue for young people.

**Table 4:** What to ask when exploring online activities

**Topics to always ask about:**	
***Activities and content viewed***	
• Gaming online	
• Social Media use (generating or browsing content)	
• Use of crisis services	
• Chatting to others with shared experience of mental health (e.g. via chat-rooms/ forums)	
• Use of apps	
• Viewing self-harm/ suicide-related content (e.g. methods and images)	
• Viewing graphic violence (eg. images/ videos of death or serious injury)	
• One-to-one online friendships	
***Online experiences***	
• Cyberbullying	
• Being groomed	
• Radicalisation	
• ‘Doxing’—having personal information shared without consent (eg. intimate images)	
***Patterns of use/ activity***	
• Frequency	
• Time spent online (eg. browsing)	
• Times of the day spent online	
• Changing use (e.g. peaks, dips, increases)	
• Impact on sleep	
**Topics to ask young people:**	
***with disordered eating***	***with self-harm or suicidal thoughts***
• Visiting ‘pro-ana’ websites	• Looking up methods of harm/ suicide
• Use of exercise apps	• Viewing images of self-harm
• Use of dieting apps	• Joining forums to discuss self-harm
• Online purchase of weight loss medicine	• Posting images of own self-harm
• Obsessively viewing food-related sites	• Visiting pro self-harm/ suicide sites
• Use of physical activity/ smart devices	• Individuals/ influencers followed
	• Consuming media with themes of depression

**Table 5:** Topics to explore to encourage reflection and self-awareness

Topics to explore to encourage reflection and self-awareness:
• how online activities impact upon how the young’s persons mental well-being (including mood, symptoms and behaviour) and self-esteem (including identity and self-image) in both positive and negative ways
• the thoughts, emotions or motivations underlying problematic behaviours online
• why particular content/ online interactions are upsetting
• how online activities impact on offline relationships
• how young people may harm others online, intentionally or unintentionally

With respect to outcomes, both panels agreed concerning activity should be flagged in clinical notes (PP 100%, YPP 90%) and that strategies for addressing online safety should be incorporated when writing personalised treatment or safety plans as detailed in Table 6 (consensus on items ranging from 75–100%). Panels agreed practitioners should encourage young people to actively manage their own online safety (PP 100%, YPP 90%) and support positive engagement with the online world instead of suggesting bans (PP 89%, YPP 95%), though local safeguarding procedures should be followed where indicated. Where recommending apps, panels expressed a need for clinicians to support their recommendations by presenting a choice of apps, offering to demonstrate how they work and following-up on use in subsequent appointments to explore effectiveness.

**Table 6:** Aspects of online safety to include within treatment/ safety plans

Personalised treatment/ safety plans should include:
• Strategies for reducing exposure to harmful content
• Strategies for recognising where patterns of online activity indicate worsening mental health
• Strategies for dealing with harmful or upsetting content
• Identification of offline alternatives to online activities
• Signposting to useful sites or apps

### Excluded statements: ‘conflicts’ and ‘near-misses’

Thirty-nine statements were excluded. These represented unclear opinion (*n* = 21), conflicts (consensus in one panel, other panel expressing opposing trend) (*n* = 4), or ‘near-miss’ statements (one panel reaching consensus, the second expressing trend in the same direction) (*n* = 14). There were no instances where both panels rejected (disagreed by consensus) the same statement, and thus no negative items for inclusion in the GPIs.

Conflicts surrounded if ‘it is acceptable for *any* clinician to ask about online activities’ (PP 95% agree, YPP 50% disagree); and if ‘it is inappropriate to ask young people who are acutely very unwell’ (low conflict, PP 53% disagree; YPP 81% agree), or ‘experiencing paranoia’ (mid conflict; PP 80% disagree, YPP 60% agree). Involvement of parents was another area of contention: 87% of clinicians thought parents should be involved where the young person is aged 12–15, while YPP opinion was split. Finally, the YPP largely disagreed with the statement ‘young people should be asked about online activities at every consultation’ (81% disagreement) while the PP were undecided (20% agree, 47% disagree).

There were 8 ‘near-miss agree’ and 6 ‘near-miss disagree’ statements (Table 7). Three referred to clinician knowledge (discussed below but not included within the GPIs). Near-miss agree items indicated other possible topics (‘what’) to cover within conversations: details of online conversations where worrying use is apparent (low agreement); viewing and posting images of bodies (medium agreement) and influencers followed (medium agreement) for patients with disordered eating; and online purchasing of items associated with harm (means) for patients with self-harm/ suicidal feelings (medium agreement). Near-miss disagree statements contributed to debate around the inappropriateness of asking some young people about their online activities, revealing a majority view that practitioners should *not* avoid asking those who are actively suicidal (PP 93%, YPP 56%), or under 10 years (100% PP, 56% YPP), and that it is acceptable for practitioners to ask about online activities where not raised by the young person (95% PP, 56% YPP). The use of pre-set questions was commonly rejected (75% PP, 58% YPP).

**Table 7:** ‘Near-miss’ statements

**Domain**	**Statement**	**Round introduced**	**Panel with consensus**^**a**^	**Trend and strength of agreement/ disagreement** ^b^
Who/ When	There are some young people who should not be asked about their online behaviour	1	PP	Disagree, Medium
Who/ When	Parents/ carers should be asked about a young person’s online activities	1	PP	Agree, Low
Who/ when	Clinicians should ask young people about their online activities only where the young person raises this	1	PP	Disagree, Low
Who/ when	It is inappropriate to ask a young person about their online activities if the young person is actively suicidal	3	PP	Disagree, Low
Who/ when	It is inappropriate to ask a young person about their online activities if the young person is under 10 years	3	PP	Disagree, Low
How	Conversations should be started using pre-set questions with everyone asked in the same way	1	PP	Disagree, Low
How	When discussing online activities, clinicians should be experts about the online world	1	PP	Disagree, Low
How	When discussing online activities, clinicians should be familiar with basic online slang	3	PP	Agree, Medium
How	When discussing online activities clinicians should be familiar with harmful content circulating	3	YPP	Agree, High
What	Discussions about worrying activity should usually include asking for details of conversations the young person has had online	1	PP	Agree, Low
What	Tailoring questions for young people with disordered eating should include asking about viewing/ posting images of bodies	3	PP	Agree, Medium
What	Tailoring questions for young people with disordered eating should include asking about specific individuals/ influencers followed	3	PP	Agree, Medium
What	Tailoring questions for young people with self-harm/ suicidal feelings should include asking about purchasing methods/ tools online	3	PP	Agree, Medium
Outcomes	When recommending apps, clinicians should offer written information about the app	2	YPP	Agree, Medium

^**a**^*PP* Practitioner panel, *YPP* Young person panel

^b^Majority view across panels and strength of agreement/ disagreement of majority view in panel without consensus: Low = 50–59%; Medium = 60- 69%; High = 70–74%

### Qualitative themes

Open-ended questions provided insight into key areas of discord and suggestions about how some GPIs could be operationalised in practice.

#### ‘Who’: which clinicians should ask?

Half of the YPP thought asking about online activities should only take place within the context of a trusting, on-going relationship where good rapport had already been established. They warned that if *any* clinician asks, this could result in a young person being asked multiple times and might exacerbate feelings of judgement, invasion of privacy, and resentment at an inappropriate level of blame being focused on online activities where other factors may be more pertinent. The PP acknowledged that an established relationship was desirable but did not regard this as a prerequisite. Their overriding perspective was that discussing online activity is important to assessment and safeguarding, so is “everyone’s responsibility” and should be addressed where possible, including in A&E:

Given online activities can be harmful and expose young people to various risks, I think it should be acceptable for any [clinician] to ask. You never know, you might be the first person to raise it. (PP)

There may be clinicians or settings that it would be more appropriate, e.g. an ongoing relationship… However, opportunities to explore this should not be wasted – if a young person… is only likely to be meeting with clinicians on a short-term or one-off basis, the opportunity to discuss online activity should be used. (PP)

#### ‘Who’: which young people—risk of asking

Members of the YPP discussed the potential inappropriateness of practitioners asking high-risk or reluctant young patients about their online use, voicing concerns that this could exacerbate symptoms, provoke distress or resentment, or lead the unexposed to harmful content:

If a person is paranoid then they may get more paranoid if asked about their online activity. (YPP)

[It is inappropriate to ask] when they are clearly hesitant and reserved about sharing. It may be a trigger for them, so the subject should be broached carefully, and perhaps only when the young person brings it up in their own time. (YPP)

Being introduced to the idea of using online activities in different ways could lead to more young people discovering new methods of self-sabotage. (YPP)

Some flagged such risk could be particularly pertinent for patients with presenting problems where issues of illness identity and competition can be relevant:

In terms of specifically eating disorders, where there is a high rate of competition to be the ‘most unwell’, giving [online] suggestions… is an indicator that that’s what other ‘anorexics’ do, therefore this is something they should be doing (YPP).

While a few practitioners acknowledged possible risk and that occasionally there could be *“better times”* to ask (for example, if a patient was acutely psychotic and needed stabilising), the general view was that avoidance could pose greater risk, so asking should not be ruled out for any particular situation. Some YPP members also presented these arguments.

A number of the scenarios described [in Delphi statements] potentially increase risk when coupled with online activity of certain kinds (e.g. suicidality) and so it would be pertinent to ensure this is accounted for within assessment (PP)

Online activity can often provide a window into what is going on for the young person, therefore avoiding it is neglecting information (PP)

Practitioners argued that risk can be mitigated by method of asking. Specifically, by a broad approach of *“curious questioning as opposed to explicitly listing harmful resources”* and being led by the young person’s answers to avoid suggestion. For instance*:*

I will avoid asking about use of specific sites or tags because I don’t want to introduce new sources of inspiration. But that wouldn’t stop me from asking about their experience and what sites they visit/ what searches they use. (PP)

Members of the YPP agreed a way forward was to invite young people to talk about what they had encountered rather than asking if they had accessed specific content. Careful consideration of the ‘how’ and ‘what’ domains was therefore seen to override concerns about ‘who’ or ‘when’.

#### ‘How’: Normalising online activities.

To support the GPI that practitioners should normalise online activities when discussing these, participants were asked for examples of how this could be done. Common ideas are summarised in Table 8, though tensions arose with respect to how a practitioner should position themselves. For example, opinion was divided about whether practitioners should attempt to use online slang. Also, while one practitioner drew on a recurring theme about the need to *“show curiosity and interest to learn”,* a YPP member warned that clinicians should *“not appear to be fascinated”* if normalisation is to be achieved, revealing a possible tension in how this could be interpreted.

**Table 8:** Free-text suggestions for supporting conversations about online activity

**Suggested methods for normalising online activity**	
• Casual reference to other young people the clinician knows or works with, or the clinician’s own use, acknowledging that the online world is central to most people’s experience (both panels)	
• Begin by asking about *‘benign, day-to-day use, such as favourite Tik-Tok dances or favourite socials ‘* (PP)	
• Begin questions about harmful use with reassurance, for instance *“sometimes when [young people] are struggling a lot, our brains seek out that kind of website”* (YPP)	
• Frame questions to openly accept the young person is online, thereby inviting them to talk about ‘which’ and ‘how’ rather than ‘whether’ (Both panels)	
• *‘Talk casually’* and ask about online activity alongside normal topics such as school or sleep so it appears equally ‘*commonplace’* and *‘not a massive deal’* (YPP)	
• Demonstrate knowledge of popular sites, platforms, activities and language/ terminology	
• Show understanding that the online world is fundamental to young people’s lives, can be good, and cannot merely be *‘cut off’* (PP)	
**Methods for supporting and empowering positive online activity**	
**Safe online practice and behavioural tactics to:** • **reduce usage and exposure** • **provide strategies for coping with harmful content**	• Use of online safety tools: reporting; blocking; use of filters to ‘clean’ feed and hide triggers (incl. trigger words and hash tags) • Use of safety apps to monitor and restrict use • Self-imposed time limits to reduce scrolling • Self-imposed screen breaks at specific times (e.g. at night, when more vulnerable/ likely to impact on sleep) • Self-imposed breaks from particular platforms • Unfollow accounts that trigger negative feelings or behaviours, including self-comparison • Limit communications to positive interactions and manage pressure to respond • Bookmark links to online help sites, resources, services and apps • Close and re-make social media accounts
**Promote insight and self-awareness**	• Journaling and discussion to recognise and monitor impact of use: identifying what is helpful and unhelpful, challenging v. rewarding sites, risks and outcomes • Discussion around reasons for engaging with harmful/ inappropriate content and how use can be problematic • Discussion to reflect on online identity • Education, e.g. around online harms, algorithms
**Signpost/ facilitate positive online use**	• Identify and focus on current beneficial uses (including supportive communities, sites and friendships) • Recommend ‘healthy’ content (e.g. positive influencers, social media promoting self-care, supportive communities) • Signpost and demonstrate positive online activities and resources (eg. support/ treatment apps, help-sites)
**Provision of offline alternatives**	• Identify offline support network • Introduce/ devise alternative offline coping strategies (e.g. don’t scroll, call a friend) and methods of distraction or self-regulation (e.g. ‘calm boxes’, mindfulness) • Build-up regular offline activities
**Practitioner-patient communications**	• Foster open, non-judgemental communication • Discuss what is upsetting online and explore patterns of behaviour and impacts in partnership • Follow-up on recommended tactics and use (incl. use of apps) • Incorporate into assessment and planning
**Help-seeking**	• Encourage discussion with trusted offline adult contacts (e.g. parents, teacher, youth worker) about online activities, especially when upset/ worried by content

#### ‘What’: Adapting conversations based on personal characteristics

It was noted that simplistic language is likely to be more appropriate for younger patients and some suggested the questions asked should take account of typical content accessed by particular age groups, such as popular apps, games or platforms. A recurring YPP concern was a potential to *“adversely influence”* younger children by leading them to harmful content if asking about some topics (e.g. pornography):

Younger people may not yet be exposed to some topics and become curious after being questioned. Filter questions. (YPP)

However, the danger of limiting discussion due to incorrect assumptions and thereby missing risky use was raised by both panels as a challenge to navigate.

I don’t think we should make assumptions about what we expect a young person’s online activity to look like. It is better to remain curious and ask everyone the same questions even if we think they may not be relevant. (PP)

Uncertainty about adapting conversations for ethnicity, gender or sexuality related to the same concern, although the YPP noted clinicians should be aware marginalised youth may experience more online hate or be more susceptible to grooming as they may more frequently look to online spaces to find relationships due to difficulties building these offline. Ideas about how to adapt for disability focused on neurodevelopmental disorders but were unclear beyond basic suggestions about accessible language.

#### Outcomes: supporting safe and positive use

To realise GPI aspirations around outcome, panels were asked for specific ideas about how practitioners can empower young people to engage positively with the online world and to suggest strategies for responding to harmful content. The YPP drew on strategies they use and find useful, while the PP shared examples of strategies they currently recommend. There was strong overlap between the responses yielded (Table 8). Participants noted these strategies are needed in combination and that facilitating insight and ability to self-monitor is fundamental to enabling young people to assert agency. For instance, prompting self-awareness of triggering content or influencers and identification of positive alternatives is a pre-requisite for commitment to behavioural strategies around blocking or unfollowing.

The YPP gave specific examples of how a practitioner could apply such strategies to address with young people the harm they could cause others online. These included: use of reflective, non-judgemental discussion to increase self-awareness about how images and other content posted by the young person may be perceived by others; advice to use tactics such as making accounts private or refraining from using hashtags in order to protect others; and recommending use of pen and paper as an alternative outlet for strong negative feelings.

#### Outcomes: involving parents/ guardians

Young people were reluctant for parents to be involved in conversations about online activity, stemming from a perception they may misunderstand online behaviour or take an *“over-exaggerated”* view leading to counterproductive actions (blocking, banning) and *“unwarranted worry or tension”* in the household. Some YPP members raised privacy, noting many young people prefer to conceal their online lives from parents and involving parents may lead to young people feeling *“ganged up on”* and practitioner-patient trust being eroded. A few practitioners similarly cautioned that involving parents should not occur without being mindful of parents’ possible misperceptions about the online world:

Some parents get very angry and alarmed…there may need to be a general conversation first with parents to assess feelings and reactions. (PP)

Practitioners however offered several ways parents could be involved in discussions where patients are under 16 years. These ranged from educating parents in general terms about the online world, to making them aware of their child’s specific online activities and included: discussing ways to support safe use, such as imposing boundaries on screen time and using parental controls; and fostering open dialogue with their child, particular around unhelpful online interactions. Some YPP members concurred that parents could be given general information about online risks and advice about supporting a young person who is “an excessive user” or “upset online” without imposing “blanket bans”. Panels stated parental involvement should be contingent upon the young person’s consent, evidence of risk, or safeguarding issues. The same ideas were expressed where the young person is over 16 years, though with greater emphasis from both panels on the young person maintaining control and there being a higher threshold of risk.

### Practitioner knowledge and support needs

Views on practitioners training and support needs were also explored following consensus that practitioners should display up-to-date language and knowledge. This was seen as essential for reassuring young people to disclose and ensuring conversations are useful. Qualitative data suggested this meant being ‘broadly familiar’ with the main social media platforms and how these function, potentially harmful mental health-related online communities, helpful sites, apps and safety features including time limiters and privacy settings, and basic online slang and text speak. Practitioners perceived a particular need to be updated about new trends given the rapidity with which the online world evolves but young people asserted that practitioners should receive more foundational training to instil appreciation of the place of the online world within a young person’s life and aid recognition of what is ‘normal’ and when to pathologize. Notably, all participants from both panels agreed that young people should play a key role in training clinicians.

## Discussion

Using a mixed-methods Delphi approach, we sought the expertise of mental health practitioners and young people with lived experience of mental health difficulties to develop good practice indicators (GPIs) around how to conduct conversations with young people about their online activities. This resulted in 27 indicators across four domains, generated from statements reaching a consensus agreement of 75% or more in both panels. These covered: i) ‘who’ and ‘when’ to ask; ii) ‘how’ conversations should be conducted; iii) ‘what’ should be discussed; and iv) ‘outcome’—how conversations should be taken forward.

There was consensus that all young people should be asked about their online activities, this should occur at first meeting and then at regular intervals, and that there are red flags indicating times where a conversation may be particularly indicated. A fluid approach was emphasised, with open-ended prompts embedded spontaneously within the flow of conversation. The importance of providing context and reassurance, normalising both online activity and discussion of this, and acknowledging positive aspects of online use to ensure a non-judgemental exchange were also highlighted. Key topics to *always* address were delineated, with additional topics to ask patients with disordered eating or self-harm behaviours, and follow-on areas for gaining more specific information about worrying activity. There was strong agreement that asking about online activity should move beyond information gathering to take the form of a deeper conversation with a focus on encouraging the individual to reflect on the meanings and impact of their online behaviour. It was agreed the primary objective should be to empowering young people with insight and strategies to actively manage their online safety and derive positive benefits, rather than on ‘stopping’ online activity. It was also agreed that concerning online activity should be flagged in clinical records and followed up, and that personalised strategies for on online safety should feature within treatment plans. Qualitative data suggested methods for normalising discussion of online activities and for supporting positive online behaviour.

Areas of conflict and uncertainty between panels surrounded possible risks of initiating conversations about online harms and whether practitioners should involve parents in discussions. Young people tended to recommend a more cautious approach, expressing concerns about the potential to exacerbate symptoms or flag the existence of risky online content if raising online activity with the very ill or very young. However, practitioners suggested the risks of not enquiring may be greater and that concern could be mitigated by method of asking. In accordance with principles of good clinical care, both panels stated parental involvement should be contingent upon the young person’s consent and/ or sufficient safeguarding concern. This was reflected by the GPIs that there should be explicit discussion around confidentiality and that all young people should have the opportunity to discuss online activity without parents being present. Involvement of parents for those under 12 was agreed within this context and implies conversation about online activity with general advice for this age group, not that confidentiality should be broken. Young people however were notably reluctant to endorse parental involvement for those over 12 years and set a high threshold of risk to be present before confidentiality should be overridden. Both panels implied parental education around online harms may be needed to ensure that involving them is supportive and productive.

The relevance of digital technology to young people’s mental health is indisputable, having been described as ‘infrastructural’ to their everyday life [16], and recognised as both as a positive and negative force. It has been suggested that practitioners address this during consultations [11] and that asking about online use could facilitate risk assessment [12]. However, to date, guidance and training about how to conduct and focus such conversations has been lacking [17]. Our good practice indicators fill this gap, offering some clarity on how practitioners might work with young people to improve online safety. While not contradicting earlier suggestions, our findings urge caution about practitioner reliance on promoting use reduction or parental involvement [1]. Our findings focus primarily on a need for open, non-judgemental exchange and aiming to increase young people’s autonomy around their online behaviour. This is in line with other evidence about the most effective consulting contexts for improving communication with adolescents [18] and available evidence about best approaches for reducing online risk [19]. Our findings also highlight a role in assisting young people to capitalise on positive aspects of the online world in relation to mental health, emphasised by some commentators. Previously expressed practitioner concern that asking about online behaviour could create risk [12] re-emerged in our young people panel but was disputed by practitioners.

### Strengths and limitations

The involvement of two panels – young people and practitioners—and use of an iterative approach underpinned by qualitative enquiry, afforded creation of a refined set of GPIs, responsive to the priorities and experiences of the major stakeholders and thus likely to have good acceptability. These are supported by a narrative providing additional nuance around some issues and including suggestions about how general statements may be operationalised in practice. To our knowledge, this is the first guidance in this area.

All panel members had relevant expertise and the study sample size, retention rate and consensus cut-points were within recommended ranges for robust Delphi methods [15]. The two panels were balanced, giving equal weight to each perspective. Our young people panel was diverse with respect to age and diagnosis. Similarly, a range of practitioners was involved with respect to clinical experience and role. However, there were limitations to inclusivity, which should be explored in further research. Males were substantially underrepresented in both panels and ethnicity and socioeconomic status were not recorded. All panel members were based in England or Wales. Our panel did not include children under 13 years, limiting our ability to reach conclusions about application for the youngest age groups. Further, inclusion of social care practitioners, may have added an additional perspective on safeguarding.

Some topics, such as young people’s use of online pornography and online gambling, were not included in the GPIs despite indication that these online activities are relevant to young people’s mental wellbeing [20, 21]. Such activities are patterned by characteristics including sex and the underrepresentation of male participants in our panel may have led to an underestimation of importance. A more diverse panel may have provided further clarification around discussion of such topics and their exclusion from the GPI topics to *always* ask about should not preclude discussion or detract from practitioners’ awareness of their relevance. Similarly, findings were less clear around personalisation of conversations for population groups, such as those with neurodevelopmental disorders, despite a sense that this was important. This may be because our panels lacked lived-experience in these areas. Finally, inclusion of a parent/ carer panel would have been informative, as highlighted by contention over when and how to involve parents in conversations. This is an area for further research.

## Conclusions

This study provides much needed preliminary good practice indicators for mental health practitioners about how to engage young people in conversations about their online activities and respond to the issues raised. Unlike traditional approaches to online harms, which have tended to focus on restriction, findings from this study promote the idea of practitioners facilitating awareness and empowering young people to exercise agency in relation to their online behaviours and safety.

Pilot work is required to examine use of the GPIs in practice. As well as considering their transferability to settings such as primary care, and to particular population groups, this should focus on examining feasibility in the context of clinical constraints relating to time and resources. It should be recognised that it may not be achievable to cover all GPIs in an initial assessment and that discussion may span subsequent sessions. The conversations proposed may be more suited to long-term clinical relationships. Further research may be able to derive a refined list of key content for initial or one-off encounters. Evaluation should also include an attempt to measure patient outcomes arising from the opportunity to address online behaviour with a practitioner and investigate which of the suggested practitioner responses appear most beneficial. Other useful next steps may include co-design of strategies and tools that may be used in crisis planning and in brief interventions to facilitate empowerment and positive use. Additionally, findings have identified potential training needs for practitioners, though this presents challenges. Consideration is required of how these are best met, where this can be focused on general principles, and the extent to which knowledge of up-to-date trends and terminology is attainable given the rapidly evolving online terrain. Education of parents/ carers may also be required if they are to be included within conversations.

## Supplementary Information

**Additional file 1.** Engagement research and questionnaire development. Describes methods used to generate the round 1 Delphi questionnaire and the underlying basis for the content.

**Additional file 2.** Full quantitative data set for Delphi study investigating how Mental Health Practitioners should converse with young people about online activities. All statements are detailed in full, showing round introduced and percentage agreement in each panel and across rounds (Tables S1-S4).

**Additional file 3.** Statements accepted, rejected and without consensus by domain, round and panel for Delphi study investigating how Mental Health Practitioners should converse with young people about online activities. The number of statements reaching consensus or being rejected in each round are summarised (Table S5) and the patterns, similarities and differences across panels are discussed.

## Data Availability

The quantitative dataset supporting the conclusions of this article are included within the article and its additional files. The qualitative dataset used and analysed during the current study are available from the corresponding author on reasonable request.

## Abbreviations

GPI: Good practice indicator
PP: Practitioner panel
YPP: Young people panel
