# Supplementary material for: Developing good practice indicators to assist mental health practitioners to converse with young people about their online activities and impact on mental health: a two-panel mixed-methods Delphi study
Source: BMC Psychiatry. 2022 Jul 19;22:485. doi: 10.1186/s12888-022-04093-w (PMC9297563; doi:10.1186/s12888-022-04093-w)
Supplement: Supplementary file 1 — Additional file 1. Engagement research and questionnaire development. Describes methods used to generate the round 1 Delphi questionnaire and the underlying basis for the content. [file 12888_2022_4093_MOESM1_ESM.docx]

**Biddle et al Additional file 1: Engagement research and questionnaire development**

Engagement research was conducted with mental health practitioners and young people as part of our broader project about the relevance of digital technology to young people’s mental health. This took the form of:

- two online surveys collecting quantitative and free-text data (the first, open to health and social care practitioners who support children and young people with their mental health, n= 99; the second, open to all young people aged 13-24years, n=268);
- two practitioner focus groups (7 participants in total, all currently providing mental health support to children or young people with a range of 5-45 years’ experience and in a variety of roles including psychiatrist, psychologist, mental health nurse and social worker);
- three young people focus groups (11 participants in total, age range x-y, all self-reporting lived-experience of mental health problems and experience of accessing support for their mental health)
- eight semi-structured practitioner interviews (all currently providing mental health support to children or young people and with a range of characteristics as with focus group participants above).

Survey participants were recruited by advertising on social media and within our pre-existing networks across the third sector and relevant professional groups. Practitioners and young people taking part in follow-up qualitative research were recruited through the same channels and also from the pool of survey participants. Issues relating to the discussion of online activities within mental health consultations formed a section in the surveys and a topic for open-ended exploration in the focus group/ interview topic guides. An exploratory approach was taken to identify key area to include in the Delphi, as well as to sense-check that the topic was important to practitioners and young people.

All relevant data were extracted and analysed using descriptive statistics or qualitative thematic coding as appropriate. For the qualitative coding, team members with differing backgrounds (LB, JD, RRK) independently reviewed the data, listing all the relevant messages they identified as emerging, such as claims, concerns, experiences, questions, stated preferences, and perceptions of good practice. JD, LB, RRK met to compare lists. Issues identified by only one team member were discussed and added, if considered by the whole team to be important, or removed if deemed unsubstantiated or out of scope. Messages were then organised into groups according to the area of concern, resulting in six domains of interest with a set of issues relating to each. Listed issues were then reframed as statements that could be rated in terms of agreement or disagreement, or as open-ended questions where further exploration was needed, to create a questionnaire. The questionnaire was written in language understandable to both practitioners and young people, with key terms such as ‘practitioner’ and ‘digital technology’ defined at the beginning to ensure shared understanding. The draft questionnaire was shared with 3 practitioners and a member of a young person’s research advisory panel, who suggested some refinements to improve clarity.
