# Supplementary material for: Developing good practice indicators to assist mental health practitioners to converse with young people about their online activities and impact on mental health: a two-panel mixed-methods Delphi study
Source: BMC Psychiatry. 2022 Jul 19;22:485. doi: 10.1186/s12888-022-04093-w (PMC9297563; doi:10.1186/s12888-022-04093-w)
Supplement: Supplementary file 2 — Additional file 2. Full quantitative data set for Delphi study investigating how Mental Health Practitioners should converse with young people about online activities. All statements are detailed in full, showing round introduced and percentage agreement in each panel and across rounds (Tables S1-S4). [file 12888_2022_4093_MOESM2_ESM.docx]

**Biddle et al. Additional file 2: Full quantitative dataset for** **Delphi study investigating how mental health practitioners should converse with young people about online activities.**

**Table S1: ‘Who’ and ‘When’ domain – results for each statement by round and panel**

| **Statement** | **Practitioner panel**  % agree (agree/ strongly agree)  % unsure  % disagree (disagree/ strongly disagree) | | | **Young people panel**  % agree (agree/ strongly agree)  % unsure  % disagree (disagree/ strongly disagree) | | | **Outcome** |
| --- | --- | --- | --- | --- | --- | --- | --- |
|  | **Round** | | | **Round** | | |  |
|  | **1** | **2** | **3** | **1** | **2** | **3** |  |
| **Introduced Round 1:** |  |  |  |  |  |  |  |
| All young people attending a mental health consultation should be asked about their online activities. | **95**  5  0 | Consensus | Consensus | 59  32  9 | 67  19  14 | **81**  19  0 | **Progress** |
| There are some young people/ groups of young people who should not be asked about their online activities. | 5  10  **85** | Consensus | Consensus | 23  32  46 | 38  14  48 | 25  6  69 | Exclude |
| There are some young people/ groups of young people who should especially be asked about their online activities. | 60  25  15 | **100**  0  0 | Consensus | **86**  9  5 | Consensus | Consensus | **Progress** |
| Parents/ carers should be asked about a young person’s online activities. | **95**  5  0 | Consensus | Consensus | 46  27  28 | 57  19  24 | 56  13  31 | Exclude |
| It is acceptable for any clinician to ask a young person about their online activity. | **95**  5  0 | Consensus | Consensus | 50  23  27 | 62  14  24 | 38  13  50 | Exclude |
| Clinicians should ask young people about their online activities at their first meeting | **84**  17  0 | Consensus | Consensus | 58  29  14 | **76**  10  14 | Consensus | **Progress** |
| Clinicians should ask young people about their online activities at every consultation | 22  33  44 | 19  25  56 | 20  33  47 | 10  29  62 | 5  14  **81** | Consensus | Exclude |
| Clinicians should ask young people about their online activities at regular intervals. | **84**  17  0 | Consensus | Consensus | 57  33  10 | **81**  10  10 | Consensus | **Progress** |
| Clinicians should ask young people about their online activities at any time when it appears necessary. | **100**  0  0 | Consensus | Consensus | **96**  5  0 | Consensus | Consensus | **Progress** |
| Clinicians should ask young people about their online activities only where the young person raises this. | 0  6  **94** | Consensus | Consensus | 39  38  24 | 43  10  48 | 38  6  56 | Exclude |
| Asking about online activities should not be a one-off conversation | **94**  0  6 | Consensus | Consensus | **77**  19  5 | Consensus | Consensus | **Progress** |
| **Introduced Round 2:** |  |  |  |  |  | | |
| The following red flags indicate it might be necessary or helpful to ask a young person about their online activities: | | | | | | | |
| Patterns of device use (over-use, change of use, increase of notifications, parent expressed concerns, device reliance) | - | **100**  0  0 | Consensus | - | **90**  10  0 | Consensus | **Progress** |
| Secrecy over device and online activities | - | **100**  0  0 | Consensus | - | **81**  5  15 | Consensus | **Progress** |
| Negative self-image (appearance, body image, comparison with others) | - | **81**  19  0 | Consensus | - | **77**  19  5 | Consensus | **Progress** |
| Isolated (eg withdrawal from friends/ family and activities, spending more time alone) | - | **94**  6  0 | Consensus | - | **90**  5  5 | Consensus | **Progress** |
| Self-harm and suicidal thoughts (esp. changing methods of harm) | - | **94**  6  0 | Consensus | - | **76**  19  5 | Consensus | **Progress** |
| Experience or history of bullying | - | **88**  13  0 | Consensus | - | **77**  14  10 | Consensus | **Progress** |
| Sleep problems, changed pattern of sleep or excessive tiredness | - | **100**  0  0 | Consensus | - | **77**  19  5 | Consensus | **Progress** |
| Problems related to disordered eating | - | **82**  19  0 | Consensus | - | 72  19  10 | **94**  0  6 | **Progress** |
| Sudden change in presentation/ disturbance of mood or behaviour (incl. drug/ alcohol use) | - | **87**  13  0 | Consensus | - | 71  14  14 | **88**  13  0 | **Progress** |
| Risk factors or signs of child sexual exploitation | - | **100**  0  0 | Consensus | - | **95**  5  0 | Consensus | **Progress** |
| **Introduced Round 3** |  | |  |  |  |  |  |
| It would be inappropriate to ask a young person about their online activity if/ when: | | | | | | | |
| The young person is clearly reluctant to speak about what they are doing online | - | - | 7  27  67 | - | - | 57  24  19 | Exclude |
| The young person is acutely unwell (e.g. experiencing psychotic episode) | - | - | 27  20  53 | - | - | **81**  0  19 | Exclude |
| The young person is actively suicidal | - | - | 7  0  **93** | - | - | 38  6  56 | Exclude |
| The young person is experiencing paranoia | - | - | 13  7  **80** | - | - | 60  7  33 | Exclude |
| The young person is under 10 years old | - | - | 0  0  **100** | - | - | 31  13  56 | Exclude |
| No rapport has been established between clinician and young person | - | - | 27  40  34 | - | - | **75**  19  6 | Exclude |
| The clinician and young person are unlikely to have a long-term relationship | - | - | 0  0  **100** | - | - | 38  31  31 | Exclude |

**Table S2: ‘How’ domain – results for each statement by round and panel**

| **Statement** | **Practitioner panel**  % agree (agree/ strongly agree)  % unsure  % disagree (disagree/ strongly disagree) | | | **Young people panel**  % agree (agree/ strongly agree)  % unsure  % disagree (disagree/ strongly disagree) | | | **Outcome** |
| --- | --- | --- | --- | --- | --- | --- | --- |
|  | **Round** | | | **Round** | | |  |
|  | **1** | **2** | **3** | **1** | **2** | **3** |  |
| **Introduced Round 1:** |  |  |  |  |  |  |  |
| Conversations about online activity should be started spontaneously as part of the flow of the consultation | **100**  0  0 | Consensus | Consensus | **85**  10  5 | Consensus | Consensus | **Progress** |
| Conversations about online activity should be started using pre-set questions where everyone is asked in the same way. | 24  35  41 | 6  19  **75** | Consensus | 30  20  50 | 31  11  58 | Stable non-consensus | Exclude |
| Conversations about online activity should be supported by recommended prompts | **83**  17  0 | Consensus | Consensus | 60  20  20 | **95**  0  5 | Consensus | **Progress** |
| Conversations about online activity should be started by the young person | 23  39  39 | 0  0  **100** | Consensus | 60  35  5 | 58  32  11 | Stable non-consensus | Exclude |
| Online activities should be discussed in more detail than just asking basic/ surface questions. For example, about whether a young person uses social media. | **95**  0  6 | Consensus | Consensus | **90**  5  5 | Consensus | Consensus | **Progress** |
| Clinicians should always explain why they are asking a young person about their online activities. For example, by explaining that some online spaces can be harmful. | **77**  17  6 | Consensus | Consensus | **80**  5  15 | Consensus | Consensus | **Progress** |
| Online activity feels different to other topics that might be asked about during a healthcare appointment. | 17  28  56 | 19  6  **76** | Consensus | 60  25  15 | **78**  11  11 | Consensus | Exclude |
| When discussing online activity with a young person, clinicians should be a learner trying to understand more about young people and the online world | **95**  6  0 | Consensus | Consensus | **80**  5  15 | Consensus | Consensus | **Progress** |
| When discussing online activity with a young person, clinicians should be curious and ask questions | **100**  0  0 | Consensus | Consensus | **95**  5  0 | Consensus | Consensus | **Progress** |
| When discussing online activity with a young person, clinicians should be able to use up-to-date language. | 72  22  6 | **88**  13  0 | Consensus | 70  30  0 | **100**  0  0 | Consensus | **Progress** |
| When discussing online activity with a young person, clinicians should be up-to-date in their knowledge about the online world. | **89**  11  0 | Consensus | Consensus | **100**  0  0 | Consensus | Consensus | **Progress** |
| When discussing online activity with a young person, clinicians should be experts about the online world and digital technology. | 24  24  53 | 6  19  **76** | Consensus | 15  35  50 | 21  26  53 | Stable non-consensus | Exclude |
| **Introduced Round 2:** |  |  |  |  |  |  |  |
| Clinicians should ask about positive aspects of online activities before addressing the negative side | - | 56  44  0 | **100**  0  0 | - | **94**  6  0 | Consensus | **Progress** |
| Clinicians should naturally embed questions about online activities within broader conversations, (eg. friendships, hobbies, things the young person finds distressing) rather than asking this as a standalone topic. | - | **88**  6  6 | Consensus | - | **100**  0  0 | Consensus | **Progress** |
| Clinicians should give examples, statistics or anecdotes of good and bad online use to help the young person share their own experiences. | - | 25  44  31 | 29  43  29 | - | 48  21  32 | 56  6  38 | Exclude |
| All young people should be offered an opportunity to discuss their online activities without their parent/ carer being present. | - | **100**  0  0 | Consensus | - | **95**  5  0 | Consensus | **Progress** |
| It is important to normalise online activities and acknowledge how commonplace online harm can be when discussing this with young people. | - | **93**  0  7 | Consensus | - | **100**  0  0 | Consensus | **Progress** |
| A clinician should explicitly address fears of judgement, eg. by saying ‘this is not about judging or blaming but making sure you are safe’, or acknowledging that young people can unintentionally enter harmful spaces. | - | **100**  0  0 | Consensus | - | **95**  5  0 | Consensus | **Progress** |
| Clinicians should explicitly discuss confidentiality and its limits when asking questions about online activity (eg. would need to report sexual abuse). | - | **93**  7  0 | Consensus | - | **95**  5  0 | Consensus | **Progress** |
| Clinicians should always openly communicate to the young person that they understand that online activity can be beneficial | - | **93**  7  0 | Consensus | - | **100**  0  0 | Consensus | **Progress** |
| A clinician should make questions about online activities appear normal (eg. state that they ask these routinely of all young people) | - | **86**  13  0 | Consensus | - | **79**  21  0 | Consensus | **Progress** |
| **Introduced Round 3** |  | |  |  |  |  |  |
| The following represent areas that a clinician should be broadly familiar with: | | | | | | | |
| The main social media platforms used by young people and how they function | - | - | **100**  0  0 | - | - | **100**  0  0 | **Progress** |
| Potential harmful features within the main platforms | - | - | **93**  7  0 | - | - | **93**  7  0 | **Progress** |
| Use support websites/ app/ online communities for common difficulties | - | - | **100**  0  0 | - | - | **89**  6  6 | **Progress** |
| Potentially harmful online communities relating to mental health. | - | - | **100**  0  **0** | - | - | **80**  13  7 | **Progress** |
| Available online safety features (eg. time limiters, privacy settings) | - | - | **92**  8  0 | - | - | **88**  6  6 | **Progress** |
| How social media/ app use various across age group | - | - | 69  23  7 | - | - | 69  25  6 | Exclude |
| Basic online slang | - | - | **79**  14  7 | - | - | 67  20  13 | Exclude |
| Current trends in popular online spaces (eg. influencers, games, online challenges | - | - | 71  21  7 | - | - | 44  31  25 | Exclude |
| Harmful content reported to be circulating | - | - | 71  29  0 | - | - | **81**  13  6 | **Progress** |

**Table S3: ‘What’ domain – results for each statement by round and panel**

| **Statement** | **Practitioner panel**  % agree (agree/ strongly agree)  % unsure  % disagree (disagree/ strongly disagree) | | | **Young people panel**  % agree (agree/ strongly agree)  % unsure  % disagree (disagree/ strongly disagree) | | | **Outcome** |  |  |  |  |
| --- | --- | --- | --- | --- | --- | --- | --- | --- | --- | --- | --- |
|  | **Round** | | | **Round** | | |  |  |  |  |  |
|  | **1** | **2** | **3** | **1** | **2** | **3** |  |  |  |  |  |
| **Introduced Round 1:** |  |  |  |  |  |  |  |  |  |  |  |
| What the young person is DOING online: when discussing a young person’s online activity, it is important to always directly ask about: | | | | | | | |  |  |  |  |
| Getting recreational drugs online | 56  39  6 | 53  40  7 | Stable non-consensus | 45  20  35 | 44  22  33 | Stable non-consensus | Exclude |  |  |  |  |
| Gambling online | 44  50  6 | 47  47  7 | Stable non-consensus | 50  20  30 | 72  6  23 | 69  19  13 | Exclude |  |  |  |  |
| Gaming online | 56  44  0 | **80**  20  0 | Consensus | **75**  10  15 | Consensus | Consensus | **Progress** |  |  |  |  |
| Social media use – generating or browsing content and communicating with others | **84**  17  0 | Consensus | Consensus | **85**  10  5 | Consensus | Consensus | **Progress** |  |  |  |  |
| Use of crisis/ help services | 61  39  0 | **94**  7  0 | Consensus | **90**  10  0 | Consensus | Consensus | **Progress** |  |  |  |  |
| Chatting to others with shared experience online about their mental health (eg. chatrooms, forums) | 73  28  0 | **100**  0  0 | Consensus | **95**  5  0 | Consensus | Consensus | **Progress** |  |  |  |  |
| Use of the dark web | 39  56  6 | 54  40  7 | 43  43  14 | 50  15  35 | 67  11  23 | 63  13  25 | Exclude |  |  |  |  |
| Use of apps | 67  33  0 | **87**  13  0 | Consensus | **75**  20  5 | Consensus | Consensus | **Progress** |  |  |  |  |
| What the young person is VIEWING online: when discussing a young person’s online activity, it is important to always directly ask about: | | | | | | | |  |  |  |  |
| Pornography | 35  59  6 | 53  40  7 | 50  50  0 | 20  30  50 | 39  17  44 | 25  25  50 | Exclude |  |  |  |  |
| Self-harm/ suicide-related content (eg. images, methods) | 73  22  6 | **87**  7  7 | Consensus | **85**  10  5 | Consensus | Consensus | **Progress** |  |  |  |  |
| Graphic violence (eg. images, videos of death/ serious injury) | 62  33  6 | **80**  13  7 | Consensus | **90**  5  5 | Consensus | Consensus | **Progress** |  |  |  |  |
| What the young person is EXPERIENCING online: when discussing a young person’s online activity, it is important to always directly ask about: | | | | | | | |  |  |  |  |
| Cyberbullying | 73  22  6 | **93**  0  7 | Consensus | **90**  5  5 | Consensus | Consensus | **Progress** |  |  |  |  |
| Grooming/ being groomed – discussions/ relationships with older individuals, as well as sexual abuse | 73  22  6 | **93**  0  7 | Consensus | **80**  15  5 | Consensus | Consensus | **Progress** |  |  |  |  |
| Radicalisation | 62  33  6 | **87**  7  7 | Consensus | 65  30  5 | **95**  0  6 | Consensus | **Progress** |  |  |  |  |
| Having personal information shared without consent (eg. intimate images, videos, ‘doxing’) | 72  22  6 | **87**  7  7 | Consensus | **80**  15  5 | Consensus | Consensus | **Progress** |  |  |  |  |
| It is important that clinicians always ask young people about the following PATTERNS of online activity | | | | | | | |  |  |  |  |
| Frequency of use | **94**  6  0 | Consensus | Consensus | **90**  10  0 | Consensus | Consensus | **Progress** |  |  |  |  |
| Time spent online | **94**  6  0 | Consensus | Consensus | **84**  11  5 | Consensus | Consensus | **Progress** |  |  |  |  |
| Times of the day online | **94**  6  0 | Consensus | Consensus | 60  20  20 | **83**  0  17 | Consensus | **Progress** |  |  |  |  |
| Changing use (eg. peaks, dips, increases in use) | 73  28  0 | **93**  7  0 | Consensus | 55  25  20 | 72  17  11 | 88  13  0 | Exclude |  |  |  |  |
| Factors triggering worrying types of use | **89**  11  0 | Consensus | Consensus | **100**  0  0 | Consensus | Consensus | **Progress** |  |  |  |  |
| Time spent online browsing | 72  22  6 | **80**  20  0 | Consensus | 65  15  20 | **83**  0  17 | Consensus | **Progress** |  |  |  |  |
| Impact on sleep | **100**  0  0 | Consensus | Consensus | **80**  15  5 | Consensus | Consensus | **Progress** |  |  |  |  |
| The questions a clinician asks about online activity need to be adapted to fit the personal characteristics of a young person, such as their: | | | | | | | |  |  |  |  |
| Disability status | 72  11  17 | **86**  7  7 | Consensus | **80**  15  5 | Consensus | Consensus | **Progress** |  |  |  |  |
| Race and ethnicity | 56  17  28 | 64  14  21 | Stable non-consensus | 68  16  16 | 72  6  22 | Stable non-consensus | Exclude |  |  |  |  |
| Gender | 50  28  23 | 50  21  28 | Stable non-consensus | 70  20  10 | 72  17  12 | Stable non-consensus | Exclude |  |  |  |  |
| Sexuality | 56  17  28 | 57  14  28 | Stable non-consensus | 60  25  15 | 72  6  17 | 63  19  19 | Exclude |  |  |  |  |
| Problem/ diagnosis | **84**  6  11 | Consensus | Consensus | **90**  10  0 | Consensus | Consensus | **Progress** |  |  |  |  |
| Age | **89**  0  11 | Consensus | Consensus | **95**  5  0 | Consensus | Consensus | **Progress** |  |  |  |  |
| Conversations should be used as an opportunity for clinicians to encourage young person to reflect on their online activities and if/ when/ how this impacts them | **94**  6  0 | Consensus | Consensus | **90**  5  5 | Consensus | Consensus | **Progress** |  |  |  |  |
| Conversations should be used to explore: | | | | | | | |  |  |  |  |
| How online activities impact on how a young person feels about themselves (eg. self-image, sense of identity, self-esteem) | **100**  0  0 | Consensus | Consensus | **95**  5  0 | Consensus | Consensus | **Progress** |  |  |  |  |
| How online activities impact on a young person’s mental wellbeing (eg. mood, self-harm, eating disorder symptoms) | **100**  0  0 | Consensus | Consensus | **100**  0  0 | Consensus | Consensus | **Progress** |  |  |  |  |
| The thoughts, emotions or motivations underlying problematic behaviours online (eg. negatively comparing self to others on social media) | **100**  0  0 | Consensus | Consensus | **90**  5  5 | Consensus | Consensus | **Progress** |  |  |  |  |
| Why particular things are triggering/ upsetting | **95**  6  0 | Consensus | Consensus | **89**  5  5 | Consensus | Consensus | **Progress** |  |  |  |  |
| How online activities impact on offline relationships | **95**  6  0 | Consensus | Consensus | 70  20  10 | **95**  6  0 | Consensus | **Progress** |  |  |  |  |
| How online activities are helpful to the young person | **100**  0  0 | Consensus | Consensus | **90**  5  5 | Consensus | Consensus | **Progress** |  |  |  |  |
| Discussions about worrying online activity should usually including asking for the following details: | | | | | | | |  |  |  |  |
| Names of sites visited | **78**  22  0 | Consensus | Consensus | 55  15  30 | **83**  11  6 | Consensus | **Progress** |  |  |  |  |
| Descriptions of content created by the young person (eg. images, posts) | **84**  17  0 | Consensus | Consensus | 60  5  35 | 72  11  17 | **88**  13  0 | **Progress** |  |  |  |  |
| Details of the conversations and exchanges the young person has online (eg. discussion threads) | **78**  22  0 | Consensus | Consensus | 50  25  25 | 50  33  17 | Stable non-consensus | Exclude |  |  |  |  |
| Details of the searches used, including hashtags and search terms | 67  33  0 | 53  40  7 | 64  29  7 | 55  5  40 | 61  6  33 | Stable non-consensus | Exclude |  |  |  |  |
| Details of participation in groups (eg. mental health support groups, pro-ana) | **94**  6  0 | Consensus | Consensus | **80**  0  20 | Consensus | Consensus | **Progress** |  |  |  |  |
| Whether specific known harmful sites have been visited | **83**  17  0 | Consensus | Consensus | **75**  10  15 | Consensus | Consensus | **Progress** |  |  |  |  |
| **Introduced Round 2:** |  |  |  |  |  |  |  |  |  |  |  |
| When discussing a young person’s online activity, it is important to always directly ask about one-to-one online friendships | - | 73  20  7 | **79**  21  0 | - | 61  17  22 | **81**  19  0 | **Progress** |  |  |  |  |
| When discussing a young person’s online activity, it is important to always directly ask about how young people may harm others online intentionally or unintentionally | - | 67  27  7 | **79**  21  0 | - | **100**  0  0 | Consensus | **Progress** |  |  |  |  |
| Tailoring of questions for young people with disordered eating should include asking about: | | | | | | | |  |  |  |  |
| Visiting pro-ana sites | - | **100**  0  0 | Consensus | - | **78**  17  6 | Consensus | **Progress** |  |  |  |  |
| Use of exercise apps | - | **100**  0  0 | Consensus | - | **89**  11  0 | Consensus | **Progress** |  |  |  |  |
| Use of dieting apps | - | **100**  0  0 | Consensus | - | **94**  6  0 | Consensus | **Progress** |  |  |  |  |
| Tailoring of questions for young people presenting with self-harm or suicidal thoughts should include asking about: | | | | | | | |  |  |  |  |
| Looking up methods of harm/ suicide | - | **93**  7  0 | Consensus | - | **88**  11  0 | Consensus | **Progress** |  |  |  |  |
| Viewing images of self-harm | - | **93**  7  0 | Consensus | - | **83**  11  6 | Consensus | **Progress** |  |  |  |  |
| Joining forums/ chat rooms to discuss suicide/ self-harm | - | **93**  7  0 | Consensus | - | **78**  11  11 | Consensus | **Progress** |  |  |  |  |
| **Introduced Round 3** |  |  |  |  |  |  |  |  |  |  |  |
| When discussing a young person’s online activity, it is important to always ask directly about viewing survivor stories or other lived-experience content | - | - | 71  29  0 | - | - | 69  25  6 | Exclude |  |  |  |  |
| Young people with disordered eating should especially be asked about: | | | | | | | |  |  |  |  |
| Viewing or posting images of bodies (eg. ‘thinspo pictures’, models) | - | - | **93**  7  0 | - | - | 69  25  6 | Exclude |  |  |  |  |
| Specific individuals or influencers followed (eg. ‘thinspo, fitness, bodybuilders) | - | - | **93**  7  0 | - | - | 69  6  25 | Exclude |  |  |  |  |
| Purchase of weight loss medications/ aids online | - | - | **86**  14  0 | - | - | **75**  6  19 | **Progress** |  |  |  |  |
| Obsessively viewing food-related content, eg. recipes, pictures, calorie counts | - | - | **93**  7  0 | - | - | **75**  6  19 | **Progress** |  |  |  |  |
| Use of physical activity-related smart devices (eg. fitbits) | - | - | **86**  14  0 | - | - | **81**  6  13 | **Progress** |  |  |  |  |
| Young people with self-harm/ suicidal feelings should especially be asked about: | | | | | | | |  |  |  |  |
| Online pacts | - | - | 64  36  0 | - | - | 50  25  25 | Exclude |  |  |  |  |
| Posting images of own self-harm | - | - | **93**  7  0 | - | - | **75**  13  13 | **Progress** |  |  |  |  |
| Purchasing methods/ tools online (eg. sharps, drugs) | - | - | **86**  14  0 | - | - | 69  13  19 | Exclude |  |  |  |  |
| Visiting pro-self-harm or pro-suicide sites/ communities/media | - | - | **86**  14  0 | - | - | **81**  6  13 | **Progress** |  |  |  |  |
| Specific individuals or influencers followed | - | - | **86**  14  0 | - | - | **75**  6  19 | **Progress** |  |  |  |  |
| Consuming media about depression or with depressive themes | - | - | **79**  21  0 | - | - | **81**  13  6 | **Progress** |  |  |  |  |

**Table S4: ‘Outcomes’ domain – results for each statement by round and panel**

| **Statement** | **Practitioner panel**  % agree (agree/ strongly agree)  % unsure  % disagree (disagree/ strongly disagree) | | | **Young people panel**  % agree (agree/ strongly agree)  % unsure  % disagree (disagree/ strongly disagree) | | | **Outcome** |
| --- | --- | --- | --- | --- | --- | --- | --- |
|  | **Round** | | | **Round** | | |  |
|  | **1** | **2** | **3** | **1** | **2** | **3** |  |
| **Introduced Round 1:** |  |  |  |  |  |  |  |
| Online activity that causes concern should be flagged in formal notes so it can be followed-up at other appointments | **100**  0  0 | Consensus | Consensus | **90**  5  5 | Consensus | Consensus | **Progress** |
| Clinicians should encourage the young person to be active in taking care of their own online safety | **100**  0  0 | Consensus | Consensus | **90**  5  5 | Consensus | Consensus | **Progress** |
| A clinician’s role is to support the young person to engage with the online world in a more positive way rather than recommending stopping online activities | **89**  11  0 | Consensus | Consensus | **95**  5  0 | Consensus | Consensus | **Progress** |
| The following could be helpful to include in a safety/ treatment plan: | | | | | | | |
| Reducing exposure to harmful online content | **100**  0  0 | Consensus | Consensus | **95**  10  5 | Consensus | Consensus | **Progress** |
| Recognising certain patterns of online activity as a sign that mental health is dipping | **100**  0  0 | Consensus | Consensus | **100**  0  0 | Consensus | Consensus | **Progress** |
| Strategies for dealing with harmful/ triggering online content | **100**  0  0 | Consensus | Consensus | **100**  0  0 | Consensus | Consensus | **Progress** |
| Offline alternatives to online activities | **84**  17  0 | Consensus | Consensus | **95**  5  5 | Consensus | Consensus | **Progress** |
| Signposting to useful online sites/ apps | **100**  0  0 | Consensus | Consensus | **75**  10  15 | Consensus | Consensus | **Progress** |
| Clinicians should involve parents in conversations about a young person’s digital technology use if the young person is <12 years | **84**  17  0 | Consensus | Consensus | **80**  10  10 | Consensus | Consensus | **Progress** |
| Clinicians should involve parents in conversations about a young person’s digital technology use if the young person is 12-15 years | 72  22  6 | **87**  7  7 | Consensus | 45  15  40 | Explored via free-text | Consensus | Exclude |
| Clinicians should involve parents in conversations about a young person’s digital technology use if the young person is 16-17 years | 45  44  11 | 54  33  13 | Explored via free-text | 25  10  65 | Explored via free-text | Consensus | Exclude |
| Clinicians should involve parents in conversations about a young person’s digital technology use if the young person is 18+ years | 6  72  23 | 7  67  26 | Explored via free-text | 10  10  **80** | Consensus | Consensus | Exclude |
| **Introduced Round 2:** |  |  |  |  |  | | |
| When recommending apps, to ensure they are effective, it is important to: | | | | | | | |
| Always offer the young person the opportunity to see how the app works, preferably by demonstrating on a phone | - | 74  20  7 | **79**  14  7 | - | **82**  0  19 | Consensus | **Progress** |
| Offer written information on how to access and use the app | - | 53  27  20 | 64  14  21 | - | 69  13  19 | **81**  13  6 | Exclude |
| Follow-up on the recommendation to see if it has worked/ been effective | - | **93**  7  0 | Consensus | - | **94**  6  0 | Consensus | **Progress** |
| Offer several choices of app that the young person can choose from | - | **93**  7  0 | Consensus | - | **94**  6  0 | Consensus | **Progress** |
| **No items introduced in Round 3** |  | |  |  |  |  |  |
