# Supplementary material for: Developing good practice indicators to assist mental health practitioners to converse with young people about their online activities and impact on mental health: a two-panel mixed-methods Delphi study
Source: BMC Psychiatry. 2022 Jul 19;22:485. doi: 10.1186/s12888-022-04093-w (PMC9297563; doi:10.1186/s12888-022-04093-w)
Supplement: Supplementary file 3 — Additional file 3. Statements accepted, rejected and without consensus by domain, round and panel for Delphi study investigating how Mental Health Practitioners should converse with young people about online activities. The number of statements reaching consensus or being rejected in each round are summarised (Table S5) and the patterns, similarities and differences across panels are discussed. [file 12888_2022_4093_MOESM3_ESM.docx]

**Additional file 3: Statements accepted, rejected and without consensus by domain, round and panel for Delphi study investigating how Mental Health Practitioners should converse with young people about online activities**

Table S5 summarises the number of statements accepted, rejected, or without consensus by domain and panel. There was strong overlap across panels, although the PP reached consensus on a greater number of items in all domains, except ‘outcome’, and were more likely to reject items than the YPP. The YPP were initially clearer about ‘what’ should be covered in conversations; in Round 1 they reached consensus on 11/ 16 suggested topics ‘a practitioners should always ask about’, while the PP endorsed only 1 (social media use). There was clear and consistent consensus on nearly all statements relating to outcome in Round 1, where key themes centred around developing young person autonomy with respect to online safety and positive use, and incorporating aspects of online behaviour within crisis/ treatment planning.

The most contentious domain was ‘who’ and ‘when’, with discord between the two panels about whether ‘there are some groups of young people who should not be asked’. The PP favoured an inclusive approach, rejecting this statement (85% disagreement), though some panel members noted that attention should be paid to timing (‘when’) and approach (‘how’) for patients who are acutely unwell, very young, have severe learning disabilities; or where the therapeutic relationship could be eroded by asking. The YPP maintained a more cautious approach, 23%-38% across rounds agreeing with the statement and expressing concerns about possible iatrogenic risks of asking young people who are particularly distressed, younger, or defensive and reluctant to disclose. Subsequently, free-text was used to generate a list of ‘red-flags’ indicating when a conversation about online activities may be particularly pertinent, and specific statements about where it may be inappropriate. Consensus was reached about red flags but not appropriateness. Another contentious statement in this domain was whether ‘it is acceptable for *any* clinician to conduct conversations with a young person about online activities’. While clinicians had a high agreement rate (95%), at Round 3, 50% of the YPP disagreed.

The panels had conflicting opinions on whether asking about online activity is different to other topics asked about during mental health consultations (PP 76% disagreement, YPP 80% agreement – ‘how’ domain). Despite this, their thoughts on how to create an open, non-judgemental exchange coalesced, including when new statements were introduced from their free-text suggestions. Two further areas of uncertainty were whether conversations should be adapted according to characteristics of the young person (‘what’), and whether parents should be involved in conversations (‘outcome’).

**Table S5: Statements accepted, rejected and without consensus by domain and panel**

| **Domain** |  | **Practitioner panel (PP)** | | | **Young person panel (YPP)** | | | **Both panels** | | | |
| --- | --- | --- | --- | --- | --- | --- | --- | --- | --- | --- | --- |
|  | **Round (cumulative number of statements)** | **Agreed (n)** | **Rejected (n)** | **No consensus (n)** | **Agreed (n)** | **Rejected (n)** | **No consensus (n)** | **Agreed (Include)** | **Near-Miss (near consensus, exclude)** | **No consensus (exclude)** | **Conflict**  **(exclude)** |
| **Who, When** | **1 (n=11)** | 7 | 2 | 2 | 3 | 0 | 8 |  |  |  |  |
|  | **2 (n=21)** | 18 | 2 | 1 | 13 | 1 | 7 |  |  |  |  |
|  | **3 (n=28)** | 18 | 6 | 4 | 18 | 1 | 9 |  |  |  |  |
|  | **Outcome** |  |  |  |  |  |  | 16 | 5 | 4 | 3 |
| **How** | **1 (n=12)** | 7 | 0 | 5 | 6 | 0 | 6 |  |  |  |  |
|  | **2 (n=21)** | 15 | 4 | 2 | 17 | 0 | 4 |  |  |  |  |
|  | **3 (n=30)** | 22 | 4 | 4 | 23 | 0 | 7 |  |  |  |  |
|  | **Outcome** |  |  |  |  |  |  | 21 | 4 | 4 | 1 |
| **What** | **1 (n=41)** | 20 | 0 | 21 | 25 | 0 | 16 |  |  |  |  |
|  | **2 (n=49)** | 39 | 0 | 10 | 37 | 0 | 12 |  |  |  |  |
|  | **3 (n=61)** | 51 | 0 | 10 | 47 | 0 | 14 |  |  |  |  |
|  | **Outcome** |  |  |  |  |  |  | 47 | 4 | 10 | 0 |
| **Outcome** | **1 (n=12)** | 9 | 0 | 3 | 9 | 1 | 2 |  |  |  |  |
|  | **2 (n=16)** | 12 | 0 | 4 | 12 | 1 | 3 |  |  |  |  |
|  | **3 (n=16)** | 13 | 0 | 3 | 13 | 1 | 2 |  |  |  |  |
|  | **Outcomes** |  |  |  |  |  |  | 12 | 1 | 3 | 0 |
